# A novel adaptation of endoscopic optic nerve decompression in non‐traumatic optic neuropathy: A retrospective case series

**DOI:** 10.1111/coa.13961

**Published:** 2022-08-03

**Authors:** Jonathan G. F. Smith, Caroline P. Smith, Philip Weir, Brendan C. Hanna

**Affiliations:** ^1^ Department of Otolaryngology Royal Victoria Hospital Belfast Belfast UK; ^2^ Department of Otolaryngology Altnagelvin Area Hospital Derry UK; ^3^ Department of Neurosurgery Royal Victoria Hospital Belfast Belfast UK

**Key Points**

- Endoscopic optic nerve decompression is of demonstrable benefit in cases of non‐traumatic optic neuropathy.
- Visual improvement can be immediate and continue for 1 year.
- Surgical decompression can prevent visual deterioration following radiotherapy for meningioma compressing the optic nerve.
- Our technique involves posterior to anterior bony decompression of the nerve.
- There were no injuries to the optic nerve, internal carotid or orbital contents in our cohort.

## 1 INTRODUCTION

The use of the endoscopic approach in cases of non‐traumatic optic neuropathy is well documented in the literature.^1^ This approach can also be of benefit in cases of visual loss from skull base meningiomas directly compressing the nerve. Specifically, the transnasal endoscopic approach affords excellent visualisation of the orbital apex and optic canal with minimal patient morbidity.

### 1.1 Objectives

We intend to highlight the benefits of our technique (which includes a posterior to anterior approach) and discuss the role of optic nerve sheath incision by reviewing visual outcomes and complications.

## 2 METHODS

### 2.1 Ethical considerations

This was an audit of local practice and therefore did not require ethical approval. All patients involved were appropriately consented for the procedure. We used the CARE checklist when writing our report.^2^

### 2.2 Design, setting, participants and main outcome measures

Patients with imaging‐proven anterior skull base meningioma were offered endoscopic optic nerve decompression if ophthalmological examination demonstrated a relative afferent pupillary defect (RAPD) and visual field deficit, or worsening visual acuity in the affected eye. Surgery aimed to prevent further visual loss due to compression, and protect against deterioration due to radiotherapy treatments.

From October 2015 to October 2019, six patients had optic nerve decompression under the care of a multidisciplinary anterior skull base team. Five patients demonstrated both RAPD and visual field defect, with Patient 6 showing reduced visual acuity only.

We retrospectively chart reviewed each case. Operative technique and complications were noted, along with change in visual acuity and colour vision. Colour vision was assessed using Ishihara plates, with the retrospective nature of our study, and involvement of multiple clinicians, impacting the standardisation of this assessment across the patient cohort.

### 2.3 Operative technique

Our technique differs from the commonly reported approach in the literature.^3^

**FIGURE 1:**
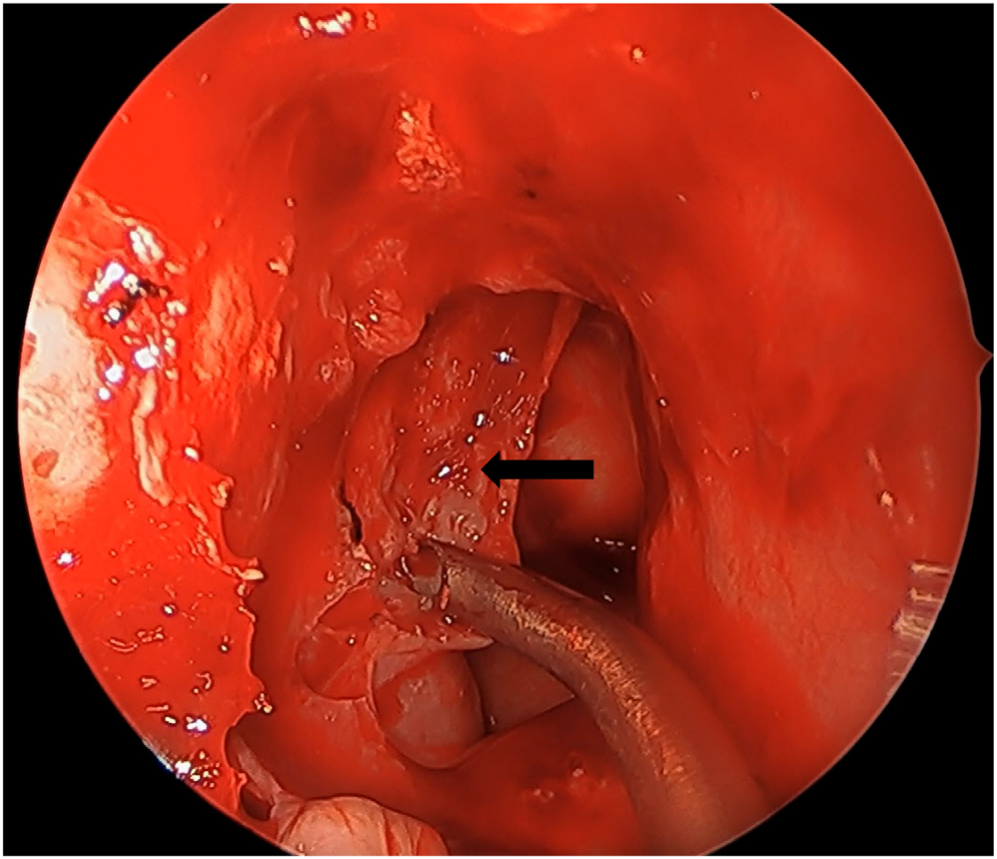
Elevation of sphenoid mucosal flap (denoted by the black arrow)

The sphenoid sinus is accessed with either a transnasal or transethmoid corridor; usually transethmoid on the side of the compressed nerve to permit dissection into the posterior ethmoid and transnasal on the opposite side for deployment of the endoscope. The transnasal corridor can be augmented if necessary with lateralisation of the middle turbinate. The lower two‐thirds of the superior turbinate is excised. A sphenoid mucosal flap is raised off the wall of the sphenoid sinus (see Figure 1): malleable instruments are required to perform this without tearing the flap in the corners.

**FIGURE 2:**
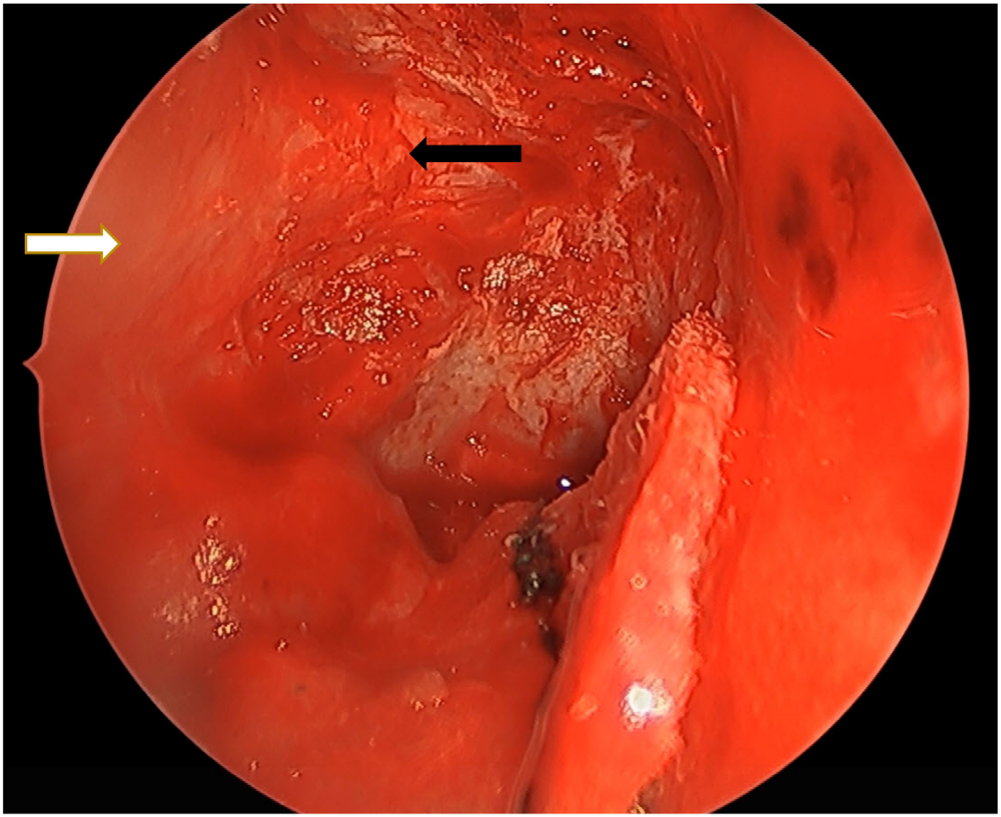
Posterior orbital periosteum (denoted by the white arrow) and optic nerve sheath (denoted by the black arrow) are visualised after bony removal.

Initially, the bone over the medial aspect of the optic canal at the level of the sphenoid is thinned using a diamond burr. The remaining bone is flaked off using a curette to allow exposure of the optic nerve sheath (see Figure 2). The dissection is then progressed anteriorly with the bone over the medial aspect of the orbital apex again thinned and excised with a curette. The periorbita is incised and the condensation of fascia representing the Annulus of Zinn is also incised medially. There can be some prolapse of orbital fat when the periorbita is incised which may otherwise obscure the dissection in the sphenoid had it not been performed first.

## 3 RESULTS

Five females and one male were included, with an average age of 51.5 years (range, 30–66 years). All six had a meningioma (see Table 1).

**TABLE 1:** Patient information

Patient	Location	Grade	Post‐decompression radiotherapy
1	Right spheno‐orbital	WHO grade I	Yes
2	Left petrous apex	WHO grade I	Yes
3	Right spheno‐orbital	WHO grade I	Yes
4	Left optic nerve sheath	N/A	Yes
5	Left optic nerve sheath	N/A	No
6	Left spheno‐orbital	WHO grade I	Yes

The average length of stay was 4.3 days (range, 1–11 days).

Six weeks post‐operatively three patients (50%) had similar visual acuity scores to baseline. Two patients (33%) had improvement, one of whom had progressed from finger counting to 6/6 vision in the initial post‐operative period. Colour vision was also noted to improve in two patients (33%) (see Table 2).

**TABLE 2:** Pre‐ and post‐operative, and post‐radiotherapy visual acuity and colour vision scoring

Patient	Visual acuity				Colour vision		Optic nerve sheath incision
	Pre‐operative	6 weeks post‐operatively	1 year post‐operatively	Post‐radiotherapy	Pre‐operative	1 year post‐operative	Performed intraoperatively?
1	6/18	6/9^a^	6/24 + 3	6/9	9/14	11/14	No
2	6/6 + 3	6/6 + 3	6/6 + 1	6/6	14/14	No change	Yes
3	6/18^a^	6/18^a^	6/18^a^	6/12	11/14	No change	Yes
4	6/60	6/60	6/36	6/36	0/14	No change	No
5	Finger counting	6/6	6/5	N/A	8/14	12/14	Yes
6	6/9	6/30^a^	6/6^a^	6/6	1/14	No change	Yes

*Note*: Column indicating the use of optic nerve sheath incision.

^a^ ‘With’ correction.

Vision deteriorated post‐operatively in one patient (17%) with acuity dropping from 6/9 to 6/30. There was an initial improvement in the immediate post‐operative period but their acuity progressively worsened over the next 4 weeks. One year after radiotherapy, visual acuity was better than the preoperative baseline.

At 1 year post‐decompression, five patients (83%) demonstrated improved visual acuity. The remaining patient dropped from 6/18 to 6/24, with an improvement in colour vision.

Of the five patients receiving adjunctive radiotherapy, four4 (80%) saw further improvement in visual acuity scores with stable vision in the fifth.

Four patients (66%) had optic nerve sheath incision to compliment bony canal decompression with one patient having an immediate intraoperative CSF leak.

In total, four patients (66%) had a CSF leak, with three noted intraoperatively (one from the anterior ethmoid region, one from the anterior fossa floor lateral to the carotid artery and the other after sheath incision) and repaired at the time using mucosal grafts. These all settled with no further intervention required.

The fourth patient developed a leak during the post‐operative recovery period and was treated with insertion of a lumbar drain.

There were no incidences of meningitis and no injuries to the internal carotid artery, optic nerve or orbital contents.

## 4 DISCUSSION

### 4.1 Synopsis of key findings

Our results for visual improvement compare favourably to the 54%–70% shown in reported literature,^3^ and show demonstrable benefit in protecting against the visual deterioration typically associated with radiotherapy.^4^

### 4.2 Comparison with other studies

A significant difference between traumatic optic neuropathy and compression from meningioma is that post‐operative radiotherapy is a frequent further stage of treatment for the latter, with studies showing local control rates of 85%–95% at 10 years.^5^ This report shows that following optic nerve decompression vision is maintained or improved during subsequent radiotherapy. The benefit of optic nerve decompression is therefore not described solely by post‐operative vision improvement as in the traumatic nerve compression group, and post‐radiation visual outcome should also be recorded to fully appreciate the benefits of this surgery.

A metanalysis by de Melo et al.^6^ reports that radiotherapy alone may improve vision in cases of optic nerve sheath meningioma; however, the location of meningioma in our cohort is more diverse. Thus, without decompression, the typical outcome of radiotherapy would be visual deterioration.^5^ Our visual outcomes post‐radiotherapy demonstrate the protective nature of prior decompression.

The surgical technique employed in this case series is different to that used in most reported literature. Berhouma et al.^3^ note the standard approach to involve initial bony decompression at the level of the lamina papyracea and note controversy around incision of the optic nerve sheath.

The use of a posterior to anterior approach, starting with drilling of the lateral sphenoid wall to identify the optic nerve canal prior to disruption of the lamina papyracea, is our preferred method of optic canal access. This prevents bulging of the periorbita that may obscure the view of the optic canal—this can be further complicated if the periorbita is torn and allows nasal herniation of orbital fat. This pragmatic “back to front” approach allows the greatest, and safest, exposure of the canal.

Incision of the optic nerve sheath step is a poorly documented, yet controversial, adjunct and we no longer routinely perform this step. A small cadaveric study by Onofrey et al.^7^ suggests that opening the optic nerve sheath may damage the nerve. Berhouma et al.^3^ also note the risk of CSF leak and ophthalmic artery injury, and after literature review, it is noted that Xu et al.^8^ similarly questioned the necessity of this step. In a cohort of patients with traumatic optic neuropathy, they found no statistically significant difference in visual outcomes between those who did and did not undergo this step. However, Thaker et al.^9^ noted the opposite, with 46% undergoing sheath incision showing improved visual outcomes versus 36% for those who did not. This finding, however, was not statistically significant and applied to traumatic cases only. We are unable to identify any published literature discussing the outcomes of optic nerve sheath incision in patients with non‐traumatic causes of optic neuropathy that would be considered analogous with our cohort.

In our cohort, the optic nerve sheath was incised with no ocular morbidity, but subsequent CSF leaks occurred. In the 50% of cases where this was identified intra‐operatively, it was effectively repaired with a simple sphenoid mucosal flap. This report, therefore, highlights that there is limited evidence for this adjunct and a high rate of CSF leak.

Behouma^3^ do not recommend formal identification or incision of the Annulus of Zinn. The endoscopic approach in the live patient does not permit definitive identification of this subtle structure. However, correlating anatomical descriptions of the structure with the location of our dissection indicates that the Annulus is divided with our incision.

## 5 CONCLUSION

In conclusion, acknowledging the small number of patients involved, this study demonstrates that endoscopic transnasal optic nerve decompression can be beneficial in cases of optic neuropathy from meningioma. We have deployed a posterior to anterior approach for optic canal dissection and have reported benefits for visual acuity. Such decompression additionally prevented further visual loss following subsequent radiotherapy.

## AUTHOR CONTRIBUTIONS

Philip Weir and Brendan C. Hanna assessed the patients and performed the surgery. Jonathan G. F. Smith, Caroline P. Smith, and Brendan C. Hanna designed the work. Jonathan G. F. Smith, Caroline P. Smith, Philip Weir, and Brendan C. Hanna analysed the data. Jonathan G. F. Smith, Caroline P. Smith, Philip Weir, Brendan C. Hanna drafted, revised and approved the manuscript. Jonathan G. F. Smith, Caroline P. Smith, Philip Weir, Brendan C. Hanna agree to be accountable for all aspects of the work.

## CONFLICT OF INTEREST

The authors declare no conflict of interest.

### PEER REVIEW

The peer review history for this article is available at https://publons.com/publon/10.1111/coa.13961.

## ETHICS STATEMENT

This was an audit of local practice and therefore did not require ethical approval.

## Data Availability

Data sharing is not applicable to this article as no new data were created or analysed in this study.

## References

[1] Lubben B , Stoll W , Grenzebach U . Optic nerve decompression in the comatose and conscious patients after trauma. Laryngoscope. 2001;111(2):320–8. 11210883 10.1097/00005537-200102000-00025

[2] Gagnier JJ , Kienle G , Altman DG , Moher D , Sox H , Riley D , et al. The CARE guidelines: consensus‐based clinical case reporting guideline development. Glob Adv Health Med. 2103;2:38–43. 10.7453/gahmj.2013.008 PMC3833570 24416692

[3] Berhouma M , Jacquesson T , Abouaf L , Vighetto A , Jouanneau E . Endoscopic endonasal optic nerve and orbital apex decompression for nontraumatic optic neuropathy: surgical nuances and review of the literature. Neurosurg Focus. 2014;37(4):E19. 10.3171/2014.7.FOCUS14303 25270138

[4] Leber K , Berglöff J , Pendl G . Dose—response tolerance of the visual pathways and cranial nerves of the cavernous sinus to stereotactic radiosurgery. J Neurosurg. 1998;88(1):43–50. 9420071 10.3171/jns.1998.88.1.0043

[5] Maclean J , Fersht N , Short S . Controversies in radiotherapy for meningioma. Clin Oncol. 2014;26(1):51–64. 10.1016/j.clon.2013.10.001 24207113

[6] de Melo L , Arruda Viani G , de Paula J . Radiotherapy for the treatment of optic nerve sheath meningioma: a systematic review and meta‐analysis. Radiother Oncol. 2021;165:135–41. 34688809 10.1016/j.radonc.2021.10.009

[7] Onofrey C , Tse D , Johnson T , Neff A , Dubovy S , Buck B , et al. Optic canal decompression: a cadaveric study of the effects of surgery. Ophthalmic Plastic Reconstruct Surg. 2007;23(4):261–6. 10.1097/IOP.0b013e3180cac220 17667093

[8] Xu R , Chen F , Zuo K , Ye X , Yang Q , Shi J , et al. Endoscopic optic nerve decompression for patients with traumatic optic neuropathy: is nerve sheath incision necessary? ORL. 2014;76(1):44–9. 24713993 10.1159/000358305

[9] Thaker A , Tandon D , Mahapatra A . Surgery for optic nerve injury: should nerve sheath incision supplement osseous decompression? Skull Base. 2009;19(4):263–71. 20046594 10.1055/s-0028-1114299 PMC2731467

